# Diagnostic challenges and outcome of fatty acid oxidation defects in a tertiary care center in Lebanon

**DOI:** 10.1186/s13023-024-03325-4

**Published:** 2024-08-29

**Authors:** Rose T. Daher, Katia El Taoum, Jinane Samaha, Pascale E. Karam

**Affiliations:** 1 Department of Pathology and Laboratory Medicine, American University of Beirut Medical Center, Beirut, Lebanon; 2 Department of Pediatrics and Adolescent Medicine, American University of Beirut Medical Center, Beirut, Lebanon; 3 Inherited Metabolic Diseases Program, Department of Pediatrics and Adolescent Medicine, American University of Beirut Medical Center, Beirut, Lebanon

**Keywords:** Fatty acid oxidation defects outcome, Neonatal screening, Middle East and North Africa

## Abstract

**Background:**

Fatty acid oxidation defects are rare autosomal recessive disorders with variable clinical manifestations and outcome. Early detection by systematic neonatal screening may improve their prognosis. Long-term outcome studies of these disorders in the Middle East and North Africa region are limited. The purpose of this study is to report the diagnostic challenges and outcome of fatty acid oxidation defects in a major tertiary care center in Lebanon, a resource-constrained country in the Middle East.

**Methods:**

A retrospective review of charts of all fatty acid oxidation defects sequential patients diagnosed and followed at our center was conducted. Collected data included: parental consanguinity, age at diagnosis, clinical presentation, biochemical profile, confirmatory diagnosis, treatment and outcome. A genotype–phenotype correlation was also performed, when available.

**Results:**

Seven types of fatty acid oxidation defects were identified in a total of 34 patients from 21 families. Most families (79%) were consanguineous (first-degree cousins). The majority were diagnosed when clinically symptomatic (78%), at various ages between 10 days and 19 years (average: 2 years). Follow-up duration spanned between 2 months and 15 years (average: 5 years). The remainder of the patients were detected while still asymptomatic by systematic neonatal screening (9%) or due to positive family history (9%). The most common defect was carnitine transporter deficiency (50%) with an exclusive cardiac presentation related to a founder variant c.981C > T, (p.Arg254*) in the *SLC22A5* gene. Medium chain acyl-CoA dehydrogenase deficiency was found in 13% only, which could be explained by the absence of systematic neonatal screening. Rare gene variants were detected in very long chain and multiple acyl-CoA dehydrogenase deficiency. The worse prognosis was observed in very long chain acyl-CoA dehydrogenase deficiency. The overall survival at last follow-up reached 75% with a complete reversal of symptoms with treatment in most patients (63%), despite their late diagnosis.

**Conclusions:**

Our experience highlights the diagnostic challenges and outcome of fatty acid oxidation defects in a resource-constrained country with high consanguinity rates. Physicians’ awareness and systematic neonatal screening are key for diagnosis. Larger genotype–phenotype studies are still needed to understand the natural history of these rare diseases and possibly improve their outcome.

**Supplementary Information:**

The online version contains supplementary material available at 10.1186/s13023-024-03325-4.

## Introduction

Fatty acid oxidation (FAO) defects are a group of rare autosomal recessive metabolic diseases caused by enzymatic deficiency of fatty acids transport, β-oxidation, or electron transfer in the mitochondria. These mainly include: (1) carnitine cycle defects: carnitine transporter defect (CTD) or primary carnitine deficiency, carnitine palmitoyl-CoA transferase-I (CPT-I), carnitine-acylcarnitine translocase (CACT), and carnitine palmitoyl-CoA transferase II (CPT-II) deficiencies, (2) β-oxidation defects: very long chain acyl-CoA dehydrogenase (VLCAD), long chain hydroxyacyl-CoA dehydrogenase (LCHAD), mitochondrial trifunctional protein (MTP), medium chain acyl-CoA dehydrogenase (MCAD) and short chain acyl-CoA dehydrogenase (SCAD) deficiencies, (3) electron transfer defects affecting electron transfer flavoprotein and electron transfer flavoprotein ubiquinone oxidoreductase, leading to multiple acyl-CoA dehydrogenase (MAD) deficiency [1]. The most common long chain FAO (LC-FAO) defects include carnitine cycle (CTD, CPT-I, CACT, CPT-II), mitochondrial β-oxidation (VLCAD, LCHAD, MTP) and electron transfer MAD deficiencies [2].

At times of prolonged fasting, fatty acids represent the main source of energy for major organs like the liver, brain, heart and skeletal muscles. Hence, most FAO defects share clinically similar presentations with various degrees of severity. These include hepatomegaly, psychomotor delay, heart failure symptoms, myalgia and exercise intolerance, while peripheral neuropathy and retinopathy may be observed specifically in LCHAD and MTP deficiencies [1]. Furthermore, life-threatening complications and even death can rapidly occur in all patients [2].

Biochemical investigations may show hypoketotic hypoglycemia, hyperammonemia, lactic acidosis, elevated liver enzymes and/or creatine phosphokinase during acute metabolic decompensations [3]. Diagnosis relies on specific abnormal patterns of blood acylcarnitine and/or urine organic acids profiles. Further confirmation can be achieved by molecular genetic testing and/or enzymatic assays [4]. Treatment is mainly preventive, based on avoidance of hypoglycemia during prolonged fasting or catabolic stress. Dietary management and supplementation with L-carnitine and/or triheptanoin and/or riboflavin are tailored depending on the FAO defect type [5].

Early recognition of these fatal disorders is crucial for preventive and timely treatment [1]. The introduction of expanded neonatal screening by Tandem Mass Spectrometry in high-income countries in the early 1990’s unveiled a highly heterogeneous incidence of FAO defects ranging between 0.9 and 15.2 per 100,000 [6]. In some Arab countries with high rates of consanguinity, like in Qatar, FAO defects incidence reaches 28/100,000 [7]. In Lebanon, in the absence of a systematic expanded neonatal screening program in the country, an estimated incidence of 6.4/100,000 was reported [8]. The importance of systematic neonatal screening for FAO defects, mainly for MCAD deficiency and some long-chain fatty acid oxidation defects was shown to improve the outcome [2, 9]. Long-term outcome studies of FAO defects are available from various high-income countries, like Canada [2], United States [10], some European countries [11], and Eastern Asia [12, 13]. However, studies from the Middle East and North Africa are scarce, with few reports from Saudi Arabia [14, 15].

The aim of this 15-year retrospective study is to report the diagnostic challenges and long-term outcome of FAO defects in a major tertiary care center in Lebanon, a resource-constrained country in the Middle East.

## Materials and methods

A retrospective chart review of all sequential patients with FAO defects followed at the American University of Beirut Medical Center, between February 2008 and February 2023, was conducted.

Collected data for each FAO defect type included initial clinical presentation, diagnosis, treatment and outcome. Clinical manifestations were categorized into cardiac, hepatic, neurological, or sudden infant death syndrome. Expanded neonatal screening and acylcarnitine profile on dried blood spots, plasma total and free carnitine levels, urine organic acids chromatography, genetic testing and/or enzyme assay on fibroblasts were all referred to established laboratories outside Lebanon. A genotype–phenotype correlation was also performed, when available. The outcome was determined based on the last clinical evaluation and classified as either asymptomatic or symptomatic with cardiac, and/or hepatic, and/or neurological complications, and/or death.

Microsoft Excel version 2208 was used for data analysis. This study was approved by the Institutional Review Board at the American University of Beirut, Lebanon.

## Results

A total of 34 patients from 21 families were diagnosed with FAO defects and followed at the American University of Beirut Medical Center, during the study period (Tables 1, 2).

**Table 1:** Diagnosis and outcome of Lebanese patients with carnitine cycle defects

Defect *Gene*	Family/CG	Patient	Age *at onset*	Initial presentation	Age *at diagnosis*	Age *at last follow-up*	Outcome	Carnitine levels (TC, FC) and Acylcarnitine profile *(µmol/L)*	Molecular diagnosis		
									*Nucleotide protein change*	*Type*	*Zygosity (classification)*
CTD *SLC22A5*	F1/ +	N1	6 m	cardiac	6 m	14y	AS	TC: 9.0, FC: 6.4	c.981C > T p.R254X	nonsense	homozygous (P)
		N2	22 m	cardiac	22 m	22 m	Death	–			
	F2/ +	N3	8 m	cardiac	8 m	16y	AS	TC: 21.0, FC: 9.0			
		N4	6 m	SID	6 m	6 m	SID	–			
	F3/ +	N5	4y	cardiac	7y	9y	AS	FC: 3.7			
	F4/-	N6	11 m	cardiac	11 m	9y	AS	TC: 19.0, FC: 10.0			
		N7	-	AS	11 m	9y	AS	TC: 12.0, FC: 7.0			
	F5/-	N8	1.5y	cardiac	1.5y	2y	AS	FC: 6.2			
		N9	2.5y	cardiac	2.5y	3y	AS	FC: 5.0			
		N10	4y	cardiac	4y	4.5y	AS	FC: 12.5			
	F6/-	N11	8 m	AS	8 m	4.7y	AS	TC: 14.3, FC: 10.7	c.539C > T p.Q180X	nonsense	homozygous (P)
		N12	8 m	cardiac	8 m	8 m	Death	–			
	F7/ +	N13	10y	cardiac	10y	14y	AS	TC: 31.2, FC: 24.7	c.64_66delTTC p.F23del	in-frame deletion	homozygous (P)
	F8/ +	N14^#^	1y	cardiac	1y	10y7m	AS	TC: 13.7, FC: 12.0, C0: 0.6	ND		
		N15^#^	-	AS*	1 m	7y	AS	TC: 10.6, FC: 8.1, C0: 1.5			
		N16^#^	-	AS*	1 m	4y	AS	TC: 24.0, FC: 18.0, C0: 3.4			
CPT-1A *CPT1-A*	F9/ +	N17	2y	hepatic	6.5y	9y	AS	C0:164, C16:0.13, C18:0.08, C0/C16 + C18:780	ND**		
	F10/ +	N18	3y	hepatic	4y	13y	AS	C0:315, C16:0.04, C18:0.02*,* C0/C16 + C18: > 3000	ND**		

CTD -carnitine transporter defect, CPT-IA-carnitine palmitoyl-CoA transferase-IA deficiency, CG-consanguinity, F-family, N-patient number, m-month, y-year, SID-sudden infant death, AS-asymptomatic, TC-plasma total carnitine, FC-plasma free carnitine, *-neonatal screening, **-enzyme assay on fibroblasts, ND-genetic testing not done, P-pathogenic, ^#^-Presumptively diagnosed. *Reference ranges (µmol/L)*: TC: 33–72, FC: 27–59, C0: 10–70, C16: 0.5–8.0, C18: 0.3–2.05, C0/C16 + C18: 0–36

**Table 2:** Diagnosis and outcome of Lebanese patients with β-oxidation and electron transfer defects

Defect *Gene*	Family/CG	Patient	Age *at onset*	Initial Presenta-tion	Age *at diagnosis*	Age *at last follow-up*	Outcome	Acylcarnitine profile *(µmol/L)*	Molecular diagnosis		
									*Nucleotide protein change*	*Type*	*Zygosity (variant classification)*
VLCAD *ACADVL*	F11/ +	N19	2d	Hepatic, Neuro	1 m	15y	Neuro, muscular, cardiac	C14:1 ↑, C16 ↑	ND**		
		N20^#^	1 m	SID	–	–	SID	–	ND		
		N21^#^	2 m	SID	–	–	SID	–	ND		
	F12/ +	N22	1 m	Cardiac	1 m	3 m	Cardiac	C14:1:1.05*,* C14:2: 0.23*,* C0:5	ND		
		N23^#^	2 m	SID	–	–	SID	–			
	F13/ +	N24	2d	Liver*	10d	3 m	Death	C14:1: 7.76, C14:2: 0.5	c.711_712delTG;c.1393A > C p.C237WfsTer15;p.N465H	frameshift/missense	compound heterozygous (P/VUS)
MTP *HADHA*	F14/ +	N25	12y	Neuro, muscular	19y	34y	Neuro, muscular	C16-OH:0.14, C18:1-OH: 0.09	c.703C > T p. R235W	missense	Homozygous (LP)
	F15/ +	N26	1y	Neuro, muscular	2y	6y	Neuro, muscular	Normal	c.955G > A p. G319S	missense	homozygous (VUS)
		N27	4 m	Neuro, muscular	9 m	9 m	Death	Normal			
MCAD *ACADM*	F16/ +	N28	2y	Hepatic	10y	21y	AS	C6:0.5, C8: 2.2, C10:1: 0.8	c.985A > G p.K329Q	missense	Homozygous (P)
		N29	-	AS*	1 m	11y	AS	C6: 0.6, C8: 3.04, C10:1: 0.4			
	F17/ +	N30	-	AS*	1 m	7y	AS	C6:1.7, C8:9.4, C10:1: 0.4			
	F18/ +	N31	-	AS*	1 m	16 m	AS	C6: 0.32, C8: 1.05	c.1084A > G p. K362Q		
SCAD *ACADS*	F19/-	N32	-	AS*	1 m	9y	AS	C4: 1.69	c.625G > A p.G209S	missense	Homozygous (B)
	F20/-	N33	-	AS*	1 m	6y	AS	C4:1.52			
MAD *ETFDH*	F21/-	N34	14y	Muscular	19y	23y	AS	C6: 0.56, C8: 0.99, C10: 1.40	c.1130 T > C; c.1529C > T p.L377P; p.P510L	missense/missense	compound heterozygous (P/LP)

VLCAD-Very long chain acyl-CoA dehydrogenase deficiency, MTP- Mitochondrial trifunctional protein deficiency, MCAD- Medium chain acyl-CoA dehydrogenase deficiency, SCAD- Short chain acyl-CoA dehydrogenase deficiency, MAD-Multiple acyl-CoA dehydrogenase deficiency, CG-consanguinity, F-family, N-patient number, d-day, m-month, y-year, Neuro-neurological, SID-sudden infant death, AS-asymptomatic, *-neonatal screening, **-enzyme assay on fibroblasts, ND-genetic testing not done, P-pathogenic, VUS-variant of unknown significance, LP-likely pathogenic, B-benign,, ^#^-Presumptively diagnosed. *Reference ranges* (µmol/L): C0: 10–70, C4: 0.09–0.8, C6: 0–0.18, C8: 0.01–0.4, C10: 0–0.36, C10:1: 0–0.13, C14:1: 0.01–0.35, C14:2: 0–0.1, C16-OH: 0–0.09, C18:1-OH: 0–0.09

**Fig. 1:**
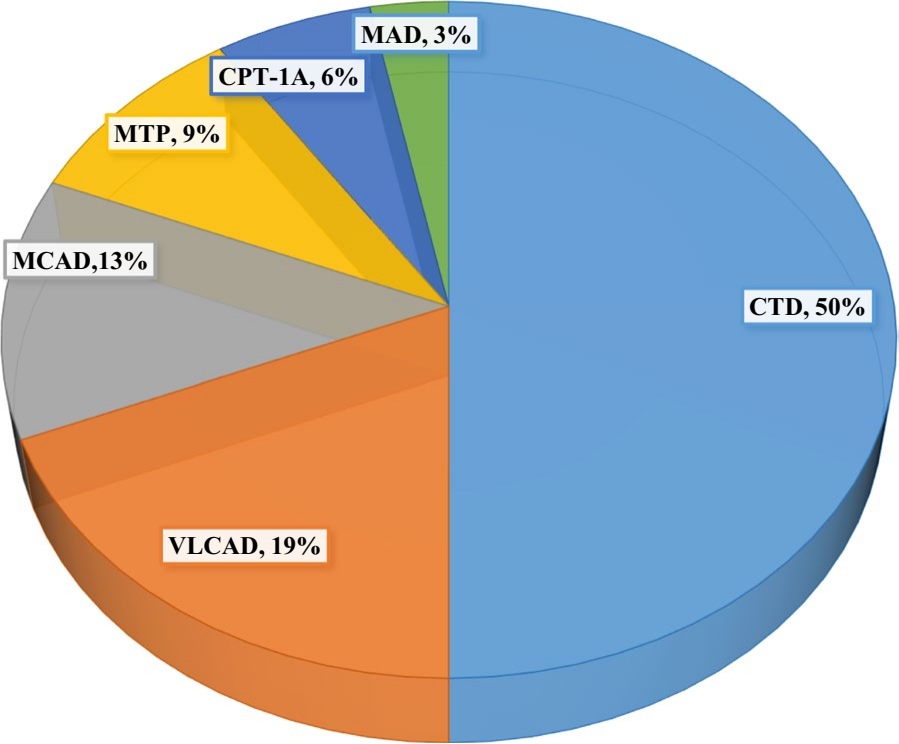
Distribution of fatty acid oxidation defects in a total of 32 Lebanese patients. CTD -carnitine transporter defect, CPT-IA-carnitine palmitoyl-CoA transferase-IA deficiency, VLCAD-Very long chain acyl-CoA dehydrogenase deficiency, MTP- Mitochondrial trifunctional protein deficiency, MCAD- Medium chain acyl-CoA dehydrogenase deficiency, SCAD- Short chain acyl-CoA dehydrogenase deficiency, MAD-Multiple acyl-CoA dehydrogenase deficiency

Seven types of FAO defects were identified: (1) carnitine cycle (CT and CPT-IA), (2) β-oxidation (VLCAD, MTP, MCAD, and SCAD), and (3) electron transfer (MAD) deficiencies (Additional file 1; Table S1). The most frequent disorder was CTD followed by VLCAD and MCAD deficiencies (Fig. 1). A few patients were detected by neonatal screening (two CTD, one VLCAD, three MCAD, and two SCAD deficiency patients). The two SCAD deficiency patients were excluded from the aggregate data, as SCAD deficiency is currently considered as a pure biochemical finding with no phenotypic expression, and its clinical relevance is controversial [16]. Among the studied 19 families, the majority were diagnosed when clinically symptomatic (78%). The age at onset varied between two days of life and 14 years (average 2 years) whereas age at diagnosis ranged from 10 days to 19 years (average: 3 years). Follow-up duration varied between two months and 15 years (average: 5 years). Most families (79%) were consanguineous (first-degree cousins). Confirmatory diagnosis was achieved in 89% of the families, by enzymatic assays in fibroblasts or by molecular testing using single gene sequencing for CTD patients and exome sequencing for the others.

Three CTD and two VLCAD deficiency patients were considered as possibly affected, based on their clinical presentation and suggestive repeatedly abnormal biochemical profiles.

The treatment was based on avoidance of prolonged fasting in all FAO types. Dietary fat restriction was recommended for patients with long chain fatty acid oxidation defects such as CPT, VLCAD, and MTP deficiencies as well as for MAD deficiency. Supplementation with oral L-carnitine and/or coenzyme Q10 and/or riboflavin was prescribed depending on each FAO defect type. The overall mortality was 25%: the highest in VLCAD deficiency reaching 67% (four out of six patients), followed by MTP deficiency in 33% (one out of three patients) and CTD in 19% (three out of 16 patients).

### Carnitine cycle defects (Table 1)

#### Carnitine transporter defect

The majority of CTD patients (75%, 12/16) presented exclusively with dilated cardiomyopathy (92%,11/12). Sudden infant death was the primary manifestation in one patient (N4). The age at onset varied from 6 months to 10 years (average 2 years 5 months). Four patients (N7, N11, N15, and N16) were identified by screening while asymptomatic, due to a positive history of affected siblings. All patients had decreased plasma total carnitine between 9.0 and 31 µmol/L (reference range: 33–72 µmol/L) and free carnitine between 3.7 and 24.7 µmol/L (reference range: 27–59 µmol/L). The diagnosis was confirmed by identification of homozygous pathogenic variants in the *SLC22A5* gene. Three patients from one family (F8) did not undergo molecular testing and were considered as possibly affected by CTD, based on their plasma carnitine levels and/or clinical presentation. All CTD patients were treated with oral L-carnitine supplementation (100 mg/kg/day in three divided doses) with complete resolution of the cardiomyopathy within six months of therapy, regardless of their age at diagnosis.

#### Carnitine palmitoyl-CoA transferase-*IA* deficiency

CPT-IA deficiency was diagnosed in two patients, at 6.5 and 4 years of age, following episodes of hypoketotic hypoglycemia and hepatomegaly, during intercurrent febrile illnesses, occurring since 2 and 3 years of age, respectively. The diagnosis was suspected on acylcarnitine profile and confirmed by enzyme assay on fibroblasts. Both patients remained asymptomatic after dietary management and recommendations to avoid prolonged fasting.

### β-oxidation defects (Table 2)

#### Very long chain Acyl-CoA dehydrogenase deficiency

VLCAD deficiency was identified in six patients (18%) from three families. The first patient (N19) from family F11 was not screened neonatally, despite a positive family history of two siblings who were lost to sudden infant death syndrome. He presented at day 2 of life with hypoketotic hypoglycemia and seizures. Echocardiography performed at day 5 of life revealed a mild biventricular hypertrophy. Qualitative acylcarnitine profile on dried blood by mass spectrometry showed highly elevated C14:1 and C16. The diagnosis was confirmed by enzyme assay on fibroblasts. He was treated with low-fat diet and medium chain triglycerides supplementation. In addition to the neurological and cardiac involvement, the patient developed recurrent episodes of rhabdomyolysis since 5 years of age requiring repeated hospitalizations. At last assessment at the age of 15 years, he had severe developmental delay, epilepsy, myopathy, and hypertrophic cardiomyopathy with no evidence of arrythmias. The 2 siblings (N20 and N21) of patient N19 were presumptively considered as suffering from VLCAD deficiency, in view of their clinical presentation with unexplained sudden infant death and the positive family history.

In the second family (F12), patient N22 was diagnosed with hypertrophic cardiomyopathy at one month of age. Family history was positive for sudden infant death at 2 months of age. Acylcarnitine profile showed elevated C14:1 and C14:2 in conjunction with a very low free carnitine level. VLCAD deficiency was suspected. Dietary treatment and L-carnitine supplementation at 50 mg/kg/day in three divided doses was initiated to normalize plasma carnitine levels. Genetic testing could not be performed, and the patient was lost to follow-up at 3 months of age.

Patient N23 was also presumptively considered to have VLCAD deficiency in view of the family history and the unexplained sudden death at 2 months of age.

Patient N24, from family F13, presented at 2 days of life with hypoketotic hypoglycemia before neonatal screening results were reported. She developed hypertrophic cardiomyopathy at one month and died at 3 months of age due to cardiac failure. Exome sequencing post-mortem revealed compound heterozygous variants (one pathogenic and one variant of unknown significance) in the *ACADVL* gene.

#### Mitochondrial trifunctional protein deficiency

MTP deficiency was identified in three patients (9%), born after uncomplicated pregnancies without signs of maternal HELLP (Hemolysis, Elevated Liver enzymes, Low Platelets) syndrome. Patient N25 had a late-onset presentation at 12 years of age, while the two other patients were symptomatic by one year of age. All three patients had the neuromyopathic phenotype. Molecular genetic testing of *HADHA* gene revealed homozygous likely pathogenic variant in F14 and a variant with conflicting classifications of pathogenicity in F15. Patients were treated with a long-chain fat-restricted diet with medium chain triglycerides supplementation and low-dose L-carnitine at 25 mg/kg/day in three divided doses to maintain normal plasma carnitine levels. In family F15, acylcarnitine profiles tested while patients were on treatment came back normal (Table 2). One patient (N27) died at 9 months of age during an intercurrent respiratory infection with rhabdomyolysis. The surviving two patients suffer from progressive myopathic deterioration and peripheral neuropathy.

#### Medium chain Acyl-CoA dehydrogenase deficiency

Three out of four patients (12%) diagnosed with MCAD deficiency were detected by neonatal screening while still asymptomatic. Interestingly, one patient (N28) had a history of undiagnosed “hepatitis” at 2 years of age, and he was retrospectively diagnosed at 10 years of age after detection by systematic neonatal screening of an affected sibling (N29). The acylcarnitine profile in all patients revealed an increase in C6, C8 and C10:1. Molecular testing by exome sequencing identified homozygous pathogenic variants in the *ACADM* gene in all patients. At last follow-up, all patients remained asymptomatic on preventive treatment (Additional file 1).

#### Short chain acyl-CoA dehydrogenase deficiency

SCAD deficiency was detected in two patients by systematic neonatal screening. Urine organic acids chromatography showed elevated excretion of ethylmalonic acid and methylsuccinic acid.

Plasma acylcarnitine profile showed elevated butyryl-isobutyryl carnitine (C4) (Table 2). Genetic testing in patient N32 detected a homozygous benign variant in *ACADS* gene. Both patients remained asymptomatic without any treatment.

### *Electron* transfer defects (Table 2)

#### Multiple acyl-CoA dehydrogenase deficiency

One patient with late-onset MAD deficiency presented at 14 years of age with progressive muscle weakness associated with episodes of acute rhabdomyolysis. Acylcarnitine profile showed increased C6, C8 and C10. The diagnosis was confirmed at 19 years of age by exome sequencing, revealing compound heterozygous pathogenic and likely pathogenic variants in the *ETFDH* gene. The patient was treated with a combination of riboflavin at 300 mg daily, L-carnitine at 50 mg/kg/day in three divided doses, and coenzyme Q10 at 200 mg daily in two divided doses. A significant improvement in the myopathy was noted within one month of initiation of therapy with no recurrence of acute rhabdomyolysis episodes at last follow-up, at 23 years of age.

## Discussion

Diagnosis and outcome of FAO disorders remain challenging with scarce data in the literature from resource-constrained countries [17]. Early detection of these defects by expanded neonatal screening has been shown to reduce mortality and morbidity rates [1, 10]. In Lebanon, despite high rates of consanguinity [18] linked to autosomal recessive disorders like fatty acid oxidation defects, neonatal screening is not mandatory and is selectively offered in some hospitals [8, 19]. Few patients (9%, 3/32) were detected by systematic neonatal screening or due to positive family history (9%, 3/32) while still asymptomatic. Usually, MCAD deficiency is reported as the most common FAO disorder detected by neonatal screening [20]. In this Lebanese series of patients, the majority (79%) were diagnosed upon clinical manifestations at various ages, in contrast to 37% (14/38) of symptomatically identified patients in Canada, for example [2]. As a result, CTD rather than MCAD deficiency was the most frequent clinically identified disorder (50% vs 13%).

Half of the patients with LC-FAO defects were still available between 5 to 15 years for follow-up, similarly to a larger study of 426 patients in the United States [17]. A shorter follow-up duration (average 2.4 years) was reported from low- to middle-income countries [6].

The overall survival in our cohort of 32 patients reached 75% at last follow-up, with a five-year survival of 53% despite the late diagnosis of most cases, in comparison to 52% in a large French pediatric cohort [21].

Genotype–phenotype correlation, when available, revealed rare variants, sometimes related to a founder effect in the highly consanguineous Lebanese population.

Interestingly, the phenotypic presentation of CTD was exclusively an isolated dilated cardiomyopathy in all cases. No muscular, hepatic or neurological symptoms were noted. The genotypic predominance of the nonsense variant, c.981C > T: (p.Arg254Ter) in the *SLC22A5* gene, already reported in three Lebanese families [22, 23] and further identified in two others in this study, is in line with a founder effect linked to this phenotypic expression. Few CTD patients (19%) were considered as possibly affected based on their clinical and biochemical profile as reported in other series [24] in the absence of genetic testing. Although most of the cases were late-diagnosed and had profoundly decreased free plasma carnitine levels, the cardiomyopathy was totally reversed following L-carnitine supplementation.

VLCAD deficiency patients were all symptomatic before one month of age with a family history of sudden infant death by 2 months of age. They exhibited the worst prognosis and the highest mortality, similar to previous reports [21]. One patient carried compound heterozygous variants in *ACADVL* gene: a pathogenic variant c.711_712delTG, (p.Cys237Trpfs*15), recently reported by Arunath et al. [25] in a South Asian patient with a similar phenotype, and a variant of unknown significance c.1393A > C, (p.Asn465His).

MTP deficiency patients had variable ages at onset with no history of maternal HELLP syndrome. Typical phenotypes were observed with chronic peripheral neuropathy in surviving patients [26, 27]. Both families carried homozygous *HADHA* missense variants confirmed by parental testing.

While acylcarnitine profile in patient N25 from family F14 was suggestive of MTP deficiency, it was normal in both patients in family F15. Acylcarnitine profiles were tested while patients N26 and N27 were on treatment, outside any metabolic decompensation. In recent reviews on fatty acid oxidation disorders, Vianey-Saban et al. (2023) [16] along with Spiekerkoetter and Vockley (2022) [28] report that acylcarnitines may be normal in patients with neuromyopathic presentation, similarly to these two siblings in family F15.

The variant c.955G > A, (p.Gly319Ser)in *HADHA* gene, identified by whole exome sequencing in family F15, was recently described as a “variant of conflicting classifications of pathogenicity” [29]. Homozygosity for this variant was confirmed by parental testing. Both parents were heterozygote carriers of the variant c.955G > A, (p.Gly319Ser) in *HADHA* gene. In addition, in silico parameters were all suggestive of a disease-causing variant:

Polymorphism Phenotyping: probably damaging, Align-GVGD (Grantham Variation Grantham Deviation): C55 (C0: least likely to interfere with function, C65: most likely to interfere with function), SIFT (Sorting Intolerant From Tolerant): deleterious, and Mutation Taster: disease causing. Furthermore, no further variant clinically relevant to the described phenotype was found. A neuropathy panel gene testing came out negative. Further clinical reports or functional studies are still needed to confirm the conflicting effect of the c.955G > A, (p.Gly319Ser) variant in *HADHA* gene.

All MCAD deficiency patients remained asymptomatic after diagnosis. The homozygous pathogenic variant c.985A > G, usually reported to cause enzymatic deficiency of less than 1% with a severe phenotype [30], did not lead to similar outcome in affected patients from two Lebanese families.

SCAD patients displayed a biochemical phenotype without any clinical expression, reflecting the benign effect of the detected variant c.625G > A, (p.Gly209Ser) in exon 6 of *ACADS* gene [31]. This variant is considered as a “susceptibility” variant, requiring other genetic or environmental factors to cause symptoms [31]. Homozygous patients for this variant may have a higher incidence of increased excretion of ethylmalonic acid [32], even though they are asymptomatic, similarly to our SCAD deficiency patients (N32, N33).

Late-onset MAD deficiency was diagnosed in one patient harboring compound heterozygous variants, c.1130 T > C; c.1529C > T, (p.Leu377Pro); (p.Leu510Pro) in the *ETFDH* gene. The previously unreported variant, c.1529C > T, (p.Leu510Pro) was considered likely pathogenic according to the American College of Medical Genetics and Genomics. Despite the lack of a clear genotype–phenotype correlation with riboflavin responsiveness [33, 34], the c.1130 T > C, (p.Leu377Pro) variant was previously described in another late-onset MAD deficiency case showing dramatical improvement upon coenzyme Q10 and riboflavin supplementation [35], like our patient.

In conclusion, our experience highlights the diagnostic challenges and outcome of FAO deficiency patients in a resource-constrained country. The outcome of other defects, mainly VLCAD remains guarded despite early detection.

CTD, the most frequently encountered FAO defect had a favorable outcome even in late-diagnosed patients. The identification of a mild variant c.981C > T, ( p.Arg254*) in the *SLC22A5* gene may explain the observed good outcome in CTD, despite the absence of systematic neonatal screening for this disorder.

In countries with limited resources like Lebanon, the implementation of systematic neonatal screening would allow earlier identification of FAO defects demonstrating a good outcome with treatment, like MCAD deficiency. Furthermore, an increased awareness among physicians of the suggestive clinical presentations of FAO defects and the appropriate diagnostic testing may allow timely recognition of these disorders. The choice of advanced biochemical testing including total and free plasma carnitine, blood acylcarnitine profile, and urine organic acids chromatography relies on the physicians’ diagnostic acumen. Molecular testing is key for an accurate diagnosis despite the cost incurred by families, in the absence of a third-party payer for such testing. Larger genotype–phenotype studies of FAO defects are still needed, especially in highly consanguineous populations. Nevertheless, performing a single gene or panel sequencing in these populations poses the difficulty of ruling out a possible dual diagnosis in the same patient. Hence, exome or even genome sequencing may overcome such limitation and confirm the diagnosis. Genotype–phenotype correlations would enable further detection and understanding of the natural history of these defects, thus tailoring the prevention and management of these rare disorders accordingly.

### Supplementary Information

**Additional file 1. Supplementary Table S1** Molecular profile of patients with fatty acid oxidation defects diagnosed and followed at a tertiary care center in Lebanon. RefSeq- Reference sequence accession number, CTD-carnitine transporter defect, VLCAD-very long chain acyl-CoA dehydrogenase deficiency, MTP- mitochondrial trifunctional protein deficiency, MCAD-medium chain acyl-CoA dehydrogenase deficiency, SCAD-short chain acyl-CoA dehydrogenase deficiency, MAD-multiple acyl-CoA dehydrogenase deficiency.

## Data Availability

Data sharing not applicable. All available data was included in the study.

## Abbreviations

FAO: Fatty acid oxidation
CTD: Carnitine transporter defect
CPT-I: Carnitine palmitoyl-CoA transferase-I
CACT: Carnitine-acylcarnitine translocase
CPT-II: Carnitine palmitoyl-CoA transferase II
VLCAD: Very long chain acyl-CoA dehydrogenase
LCHAD: Long chain hydroxyacyl-CoA dehydrogenase
MTP: Mitochondrial trifunctional protein
MCAD: Medium chain acyl-CoA dehydrogenase
SCAD: Short chain acyl-CoA dehydrogenase
MAD: Multiple acyl-CoA dehydrogenase
LC-FAO: Long chain -fatty acid oxidation
