# Supplementary material for: Diagnostic challenges and outcome of fatty acid oxidation defects in a tertiary care center in Lebanon
Source: Orphanet J Rare Dis. 2024 Aug 29;19:315. doi: 10.1186/s13023-024-03325-4 (PMC11363453; doi:10.1186/s13023-024-03325-4)
Supplement: Supplementary file 1 — Additional file 1. Supplementary Table S1 Molecular profile of patients with fatty acid oxidation defects diagnosed and followed at a tertiary care center in Lebanon. RefSeq- Reference sequence accession number, CTD-carnitine transporter defect, VLCAD-very long chain acyl-CoA dehydrogenase deficiency, MTP- mitochondrial trifunctional protein deficiency, MCAD-medium chain acyl-CoA dehydrogenase deficiency, SCAD-short chain acyl-CoA dehydrogenase deficiency, MAD-multiple acyl-CoA dehydrogenase deficiency. [file 13023_2024_3325_MOESM1_ESM.docx]

| **Defect** | **Gene: RefSeq** | **Variant** | **Effect** | **Type** | **Zygosity** | **Classification** |
| --- | --- | --- | --- | --- | --- | --- |
| CTD | *SLC22A5:* NM_003060.4 | c.981C>T | (p.Arg254*) | Nonsense | Homozygous | Pathogenic |
|  |  | c.539C>T | (p.Gln180*) | Nonsense | Homozygous | Pathogenic |
|  |  | c.64_66delTTC | (p.Phe23del) | In-frame deletion | Homozygous | Pathogenic |
| VLCAD | *ACADVL:* NM_000018.4 | c.711_712delTG;  c.1393A>C | (p.Cys237Trpfs*15);  (p.Asn465His) | Frameshift;  Missense | Compound heterozygous | Pathogenic; Variant of unknown significance |
| MTP | *HADHA*: NM_000182.5 | c.703C>T | (p.Arg235Trp) | Missense | Homozygous | Likely pathogenic |
|  |  | c.955G>A | (p.Gly319Ser) | Missense | Homozygous | Conflicting pathogenicity |
| MCAD | *ACADM:* NM_000016.6 | c.985A>G | (p.Lys329Glu) | Missense | Homozygous | Pathogenic |
|  |  | c.1084A>G | p.(Lys362Glu) | Missense | Homozygous | Pathogenic |
| SCAD | *ACADS:* NM_000017.4 | c.625G>A | (p.Gly209Ser) | Missense | Homozygous | Benign |
| MAD | *ETFDH:* NM_004453.3 | c.1130T>C; c.1529C>T | (p.Leu377Pro);  (p.Leu510Pro) | Missense; Missense | Compound heterozygous | Pathogenic; Likely pathogenic |

**Supplementary Table S1** Molecular profile of patients with fatty acid oxidation defects diagnosed and followed at a tertiary care center in Lebanon. RefSeq- Reference sequence accession number, CTD-carnitine transporter defect, VLCAD-very long chain acyl-CoA dehydrogenase deficiency, MTP- mitochondrial trifunctional protein deficiency, MCAD-medium chain acyl-CoA dehydrogenase deficiency, SCAD-short chain acyl-CoA dehydrogenase deficiency, MAD-multiple acyl-CoA dehydrogenase deficiency.
